# Medical Practice in New Mexico

**Published:** 1897-11

**Authors:** C. M. Ramsdell

**Affiliations:** Lampasas, Texas: Late of Hagerman, New Mexico


For the Texas Medical Journal.

### MEDICAL PRACTICE IN NEW MEXICO.

BY C. M. RAMSDELL, A. M., M. D., LAMPASAS, TEXAS:
Late of Hagerman, New Mexico.

HE WHO attempts to practice medicine in New Mexico will soon learn to appreciate the *vis medicatrix naturæ*, not only from its noticeable potency here, as compared with other climes, but from the fact that he will find himself so often compelled to rely almost solely upon that power for the recovery of his patients.

The writer spent one year in a portion of the territory nearly a hundred miles from any railroad or any place where medical supplies could be obtained; and many a time did he congratulate himself on having selected, for his armamentarium a line of drugs having a wide range of usefulness.

The little Mexican village of La Luz is situated at the foot of the western slope of the Sacramento mountains. It numbers about 150 inhabitants, mostly Mexicans, and these I found, to my surprise, are nearly all Methodists. The white inhabitants we found very nice, friendly people, and during two months or more that myself and family lived among them, we received many favors at their hands. The village is beautifully situated, with the great plains of San Augustine stretching away to the west for seventy miles to the San Andres mountains, and the lofty range of the Sacramentos rising abruptly from the plains only a mile or two to the east. In the middle of the great plains lie the "white sands," a stretch of pure white gypsum forty miles long and half as wide, in some places, looking like snow banks at a distance, and on close inspection the so-called sand is almost exactly like granulated sugar. The village is famed for the quantity and quality of fruit raised there, and during our stay we had all we could eat every day and all day—peaches, grapes, apples, pears, prunes, quinces, crab-apples, raspberries—to say nothing of the wild cherries, gooseberries and elderberries found in the woods on the mountains.

One day I went to see a patient twelve miles up the side of the mountains, and as night was close at hand when my visit was done, the family invited me to stay over night. Being anxious to return home, as I had promised to do, I declined the invitation and started down the mountain. Night came on before I was half way down, and with it a storm. The road was a dangerous one even in daylight, full of sharp turns and narrow places, where no two teams could pass each other, and plenty of chances for a tumble of hundreds of feet down the rocky sides of the canons. I was soon drenched to the skin, but had to go slow and feel my way in the dark. I heard the roar of the fast swelling torrent as the heavy downpour of rain began to wash in from the hillsides, and, on nearing the junction of two creeks, La Luz and Fresual, I heard a sound, new to me, but unmistakeable as to its cause, and very terrifying to me at that time, as I was not sure but that the bridge might be gone and I might have to ford the stream. It was a peculiar, popping noise caused by good-sized bowlders knocking against each other and against the rocky sides and bottom of the ravine, as they were rolled along by the force of the torrent—nice things to strike a horse's leg, eh? Luckily the bridge was safe, and where I had to ford the stream at last, at the foot of the mountain, it had a chance to spread out at the plain so that it was no longer dangerous. There is very little travel at night among the mountains, doctors and mail carriers being about the only persons who venture on it.

As winter approached we moved up near the summit of the range and located near the head of Fresual canon. These mountain people are nearly all Texans, and when that is said there is no need to add anything in regard to their hospitality or their bravery; but one particular instance of neighborly kindness I cannot forbear to mention:

One terrible December day, the darkest of my life, we sat, my wife and I, by the sick bed of our darling child. Pneumonia had attacked her, and for eleven days we had battled, as best we could, under adverse surroundings, insufficient shelter, stormy weather, a scanty supply of medicines, and no medical aid at hand; and now the end was near. We had sent word of our trouble to an old friend from Bell County, Texas, Mrs. Frank Goodsell, who lived six miles away, on the other side of the mountain, but on account of the stormy weather and the almost impassable conditions of the roads, the message did not reach her for several days. The moment she heard of our little one's illness she saddled her horse and braved the dangers of a ride through the forest in spite of the storm and fast approaching night, and came to our aid. She sat with us as we watched the tired eyelids growing heavy with the sleep of death; her gentle hands helped prepare the little form for burial, and her generous sympathy was beyond price in that time of trial. If ever my feet press the golden streets of the new Jerusalem, I am sure that no face among all the angelic throng will appear more beautiful than did hers as she entered our humble doorway at the close of that stormy winter day. Where people have to send forty or fifty miles for medical aid, it often happens that the crisis is past before the physician arrives. In such cases it is fortunate for his reputation if the turn be favorable or the case be of such a nature that the friends of the patient can judge correctly concerning it.

Some curious cases have fallen under my observation which I shall take pleasure in laying before the readers of the JOURNAL in a future article.

